# Towards a core outcome set for dysarthria after stroke: What should we measure?

**DOI:** 10.1177/02692155241231929

**Published:** 2024-02-19

**Authors:** Claire Mitchell, Kate Woodward-Nutt, Annette Dancer, Stephen Taylor, Joe Bugler, Audrey Bowen, Paul Conroy, Brooke-Mai Whelan, Sarah J Wallace, Sabrina El Kouaissi, Jamie Kirkham

**Affiliations:** 1 Division of Psychology, Communication & Human Neuroscience, Geoffrey Jefferson Brain Research Centre, 5292 University of Manchester, Manchester, UK; 2 Research and Innovation, Northern Care Alliance, Salford Royal Hospital, Salford, UK; 3 Healing, Empowering and Recovering from Dysarthria, HEARD group, Patient Public Involvement, Manchester, UK; 4 Division of Psychology & Mental Health, Geoffrey Jefferson Brain Research Centre, 5292 University of Manchester, Manchester, UK; 5 School of Linguistic, Speech and Communication Sciences, 8809 Trinity College Dublin, Dublin, Ireland; 6 School of Health and Rehabilitation Sciences, 1974 The University of Queensland, Saint Lucia, QLD, Australia; 7 Queensland Aphasia Research Centre, 1974 The University of Queensland, Brisbane, QLD, Australia; 8 Surgical Treatment and Rehabilitation Service (STARS) Education and Research Alliance, 1974 The University of Queensland and Metro North Health, Brisbane, QLD, Australia; 9 Centre for Biostatistics, 5292 University of Manchester, Manchester Academic Health Science Centre, Manchester, UK

**Keywords:** Dysarthria, stroke, outcome assessment (health care), rehabilitation

## Abstract

**Objective:**

To identify and agree on what outcome domains should be measured in research and clinical practice when working with stroke survivors who have dysarthria.

**Design:**

Delphi process, two rounds of an online survey followed by two online consensus meetings.

**Setting:**

UK and Australia.

**Participants:**

Stroke survivors with experience of dysarthria, speech and language therapists/pathologists working in stroke and communication researchers.

**Methods:**

Initial list of outcome domains generated from existing literature and with our patient and public involvement group to develop the survey. Participants completed two rounds of this survey to rate importance. Outcomes were identified as ‘in’, ‘unclear’ or ‘out’ from the second survey. All participants were invited to two consensus meetings to discuss these results followed by voting to identify critically important outcome domains for a future Core Outcome Set. All outcomes were voted on in the consensus meetings and included if 70% of meeting participants voted ‘yes’ for critically important.

**Results:**

In total, 148 surveys were fully completed, and 28 participants attended the consensus meetings. A core outcome set for dysarthria after stroke should include four outcome domains: (a) intelligibility of speech, (b) ability to participate in conversations, (c) living well with dysarthria, (d) skills and knowledge of communication partners (where relevant).

**Conclusions:**

We describe the consensus of ‘what’ speech outcomes after stroke are valued by all stakeholders including those with lived experience. We share these findings to encourage the measurement of these domains in clinical practice and research and for future research to identify ‘how’ best to measure these outcomes.

## Introduction

Dysarthria is a type of communication impairment that commonly occurs after a stroke. It presents in a variety of ways, with varying degrees of severity, but usually leads to reduced intelligibility due to impaired speech production.^1^ We know that dysarthria affects 52% of stroke survivors.^2^ Stroke survivors with dysarthria have poorer health outcomes, reduced psychological well-being and experience social isolation^3^ when compared to those with no communication difficulties.

Intervention research in dysarthria is limited, a Cochrane review found five studies for inclusion.^4^ Despite this small number of trials, there were eleven different outcome measures used and at different time points. The variety of measures used means it is difficult to compare findings from these trials, whereas combining similar data from studies would give us clearer information on what treatments are most effective. The creation of a core outcome set is urgently needed to improve quality and efficiency of future research in dysarthria to reduce the widely recognised problem of waste in medical research.^5^

A core outcome set is a standard set of outcomes that should be measured and reported in all studies related to a particular health condition.^6^ This allows research findings to be compared, combined, and contrasted and reduces reporting bias.^7^ There are many examples of core outcome sets that are in use across related stroke conditions and contribute to clinical and research practices.^8^ Of particular interest is the successful development of a core outcome set for aphasia in 2019,^9^ this language impairment also affects stroke survivors and this work is now being implemented in research and clinical practice. Challenges to implementation of core outcome sets^10^ may relate to the lack of appropriate measures, limited involvement of key stakeholders and a lack of awareness once developed.^11^

Our ultimate aim is to develop a consensus-based core outcome set for dysarthria after stroke. The aim of the present study is to agree ‘what’ outcomes of speech to measure (COS-Speech). This is the first step in developing a core outcome set for speech, and future research will determine ‘how’ these outcomes could be measured. This consensus work is being developed in partnership with key stakeholder groups: Stroke survivors, speech and language therapists/pathologists and researchers. It is anticipated that a core outcome set for dysarthria will improve consistent and relevant outcome reporting for people with dysarthria in both clinical and research settings.

## Methods

Ethics approval was granted by the University of Manchester Ethics Committee 1 2022-13303-22550 and The University of Queensland, 2022/HE000641. Methods followed the Core Outcome Set standards for development recommendations (COS-STAD) and the Core Outcome Set Standardised Protocol (COS-STAP) checklist.^12,13^ The COS-Speech study used a Delphi process and was carried out in the following five steps:

**Step 1:** A comprehensive long list of possible outcome domains was co-produced with our patient and public involvement group of three stroke survivors all with lived experience of dysarthria called Healing, Empowering And Recovering from Dysarthria (HEARD) and included the outcomes routinely used in efficacy or effectiveness trials found in the dysarthria Cochrane review.^4^ These outcomes were grouped into relevant headings of body functions, activities and participation, major life areas, environmental factors, attitudes, body structures and personal factors using the International Classification of Functioning, Disability and Health (ICF).^14^ These groupings were carried out by the HEARD group and research study team (CM, KWN, AB, PC, BMW, SJW, JK and HEARD representative) and were used to develop questions for an online survey to understand which outcome domains were important to survey participants.

**Step 2:** Participants were recruited from three stakeholder groups in the UK and Australia with the following inclusion criteria: (a) Stroke survivors with lived experience of dysarthria, (b) speech and language therapists/pathologists with experience in treating patients with dysarthria, and (c) communication researchers from any professional background. Recruitment was done through multiple methods: Online, social media advertising, the study video https://youtu.be/Axl_1MTYovQ, attendance at stroke communication groups or invitation emails sent to these groups and to networks of clinicians and researchers. All participants gave informed consent prior to participating in this study. Demographic data was only collected for the stroke survivor stakeholder group, as this was most relevant for data analysis in this short and focused study.

**Step 3:** Two rounds of an online survey. In both rounds, participants rated each question on a scale of importance with 9 being the most important and 1 being the least important. Rankings of 7–9 indicated critical importance, 4–6 were important but not critical, while 1–3 were of limited importance. Participants were invited to suggest any additional outcomes they thought were important to consider after round one, but none were added or changed following discussion by the study management groups. Of the 11 suggested additional outcomes, 4 were interventions not outcomes, 6 were already included and 1 was not a factor in dysarthria. In round 2 of the survey participants saw the aggregated results for each stakeholder group from the previous round and were reminded of their own ratings from the previous round and were asked to rate the items again.

**Step 4:** Two online COS-Speech consensus meetings were carried out following the survey rounds. These were open to all participants to attend and participants gave informed consent to participate. This was chaired by an independent facilitator, with a background in Health Research Methodology from a different institution. We collated the results of the survey by stakeholder group and organised the findings in preparation for the meeting into three groups.

- 1: ‘Consensus in’ this meant 70% of participants voting in the survey *from all three* stakeholder groups had rated this question as critically important 7–9, and fewer than 15% in each stakeholder group had scored 1–3.
- 2: ‘Consensus unclear’ this meant 70% of participants had voted this question as 7–9 in *only one or two out of the three groups*, and fewer than 15% in each stakeholder group had scored 1–3.
- 3: ‘Consensus out’ this meant *none of the three groups* had rated this as important, 50% or fewer participants scoring it 7–9 in each stakeholder group.

Consensus meeting participants then discussed (where relevant) and voted on all outcomes, this included those where consensus was ‘in’, ‘unclear’ and ‘out’ from the survey results. The consensus meetings involved discussion, debate and electronic voting in Zoom on all 70 outcomes from the Delphi survey (regardless of their rating in the surveys). We voted first on the outcomes rated as ‘consensus in’ and ‘consensus out’ as they were the clearest to decide on. More time was then given to debate the outcomes where consensus was ‘unclear’, and this allowed participants to argue for or against inclusion into the final core outcome set. To be included in the COS 70% of all participants at the meeting had to vote ‘yes’ this was critical’.

### Change from published protocol in step 4

In the published protocol^15^ we proposed using the second consensus meeting to match COS-Speech to existing measurement instruments found in the concurrent systematic review we carried out (registered on PROSPERO CRD42022302998 at https://www.crd.york.ac.uk/PROSPERO). Instead, this second meeting was needed to agree and finalise COS-Speech and how to group the outcomes.

**Step 5:** A multi-method approach to disseminating COS-Speech has been used. Reporting is done following the COS-STAR (Core Outcome Set-STandards for Reporting) guidelines.^16^

## Results

The initial long list generated 77 potential outcomes; 46 suggested by the HEARD (Healing, Empowering And Recovering from Dysarthria) group of stroke survivors and 31 from published research. The survey was created from this long list, following removal of seven which were either duplicates or not considered by the study management or HEARD group to be a valid outcome domain. The final long list of 70 was grouped into 11 outcome domains.

The 11 domains were (1) conversations with family/significant people the person with dysarthria knows very well and sees daily or frequently (e.g.: family; people you share a home with or see at least several times a week; (2) conversations with friends/acquaintances/family who are seen fairly often or regularly; (3) conversations with strangers; (4) perception of speech intelligibility; (5) everyday speaking; (6) communication partners; (7) everyday life activities; (8) psychological well-being; (9) how speech is affected; (10) the muscles involved in speaking; (11) how speech is assessed.

Recruitment and completion of the two rounds of the survey and consensus meetings are shown in Table 1. The biggest group of participants recruited for both rounds were speech and language therapists/pathologists, followed by stroke survivors, with the smallest group being researchers. We had representation of at least 2 people from each stakeholder group at both consensus meetings.

**Table 1.** Participant recruitment to Delphi survey round 1 & 2 and consensus meetings 1 & 2.

Round 1 of the Delphi online survey for COS-Speech			
	UK (none/ part / fully complete)	Australia (none/ part / fully complete)	Total (none/part complete/fully)
Stroke survivors	17(0/0/17)	12 (1/2/9)	29 (1/2/26)
Speech/language therapists/pathologists	26(2/5/19)	12(0/0/12)	38 (2/5/31)
Researchers	18 (0/0/18)	9 (0/1/8)	27 (0/1/26)
Round 2 of the Delphi online survey for COS-Speech (*including the one participant who withdrew)			
	UK (none/ part complete/fully)	Australia (none/ part complete/fully)	Total (none/ part complete/fully)
Stroke survivors	17 (4*/1/12)	12 (7/1/4)	29 (11/2/16)
Speech/language therapists/pathologists	24 (8/0/16)	12 (1/0/11)	36 (9/0/27)
Researchers	18 (2/0/16)	9 (2/1/6)	27 (4/1/22)
Attendance at consensus meeting 1/meeting 2			
	UK	Australia	Total
Stroke survivors	2/2	2/2	4/4
Speech/language therapists/pathologists	1/5	1/1	2/6
Researchers	3/3	3/3	6/6

Demographic data relating to the stroke survivor participants completing the online survey showed we had slightly more men than women complete rounds 1 and 2 (Table 2). We recruited at least one participant from each decade aged between 40 and 90 years old and three participants from a black, Asian or other minority ethnic background (11% round 1/17% round 2). Time post-stroke showed that most participants were between 5–10 years post-stroke.

**Table 2.** Demographic data for 29 stroke survivors who completed at least part of Delphi online survey round 1 & round 2.

Stroke Survivors		Round 1	Round 2
Number recruited (part completed)		29 (n = 28 used below)	18/29 from round 1 (n = 18 used below)
Men: Women: Not reported		16 (57%): 10 (36%): 2 (7%)	10 (56%): 7 (39%):1 (5%)
Age range:	41–50 years	4 (14%)	3 (17%)
	51–60 years	6 (21%)	5 (28%)
	61–70 years	9 (32%)	6 (33%)
	71–80 years	5 (18%)	2 (11%)
	81–90 years	2 (7%)	1 (5%)
	Not reported	2 (7%)	1 (5%)
Ethnic Background as self-identified and described by the stroke survivors:			
Black British British-Jamaican/Caribbean heritage		3 (11%)	3 (17%)
UK/British/English speaking/European/Australian/Caucasian Anglo-Saxon Australian		22 (79%)	13 (67%)
Not recorded		3 (11%)	2 (11%)
Time post-stroke:			
6–12 months		2 (7%)	1 (5%)
12–24 months		5 (18)	2 (7%)
2–5 years		6 (21%)	4 (23%)
5–10 years		8 (29%)	6 (33%)
> 10 years		5 (18%)	4 (23%)
Not reported		2 (7%)	1 (5%)

Thirty-four of the original 70 outcomes in round one of the survey met the threshold for inclusion in a core outcome set after round two where over 70% in each group scored 7–9 in the survey. In total, 19 were rated critical by only two stakeholder groups, 8 were rated critical by only one stakeholder group and 9 were not rated critical by any group where less than 70% of each group scored 7–9 (Supplemental Materials 1 and 2 for survey results).

Consensus meeting 1 was carried out as planned in March 2023, with consensus meeting 2 a month later facilitated by the same external facilitator (Supplemental Material 3 for aggregated survey results for discussion in meetings). In total, 40/70 outcomes were considered critical for inclusion and 30 outcomes not included in the final core outcome set (Supplemental Material 4 for the voting results). The 40 outcomes were discussed at the final meeting and grouped together which resulted in four key outcome domains: intelligibility of speech, ability to participate in conversations, living well with dysarthria and communication partner skills and knowledge, shown in Table 3. It was agreed that all four domains must consider the impact of context and environmental factors such as background noise and the emotional state of the individual. There was also discussion around ‘who’ would be measuring these domains and it was agreed that this should include clinical measures and patient-reported measure and/or family/friends reported measure.

**Table 3.** Core outcome set for speech (dysarthria after stroke).

Core Outcome Set to be used in all clinical & research activities for people with post-stroke dysarthria	
Domains:	In brief the outcomes to be measured:
1. Intelligibility of speech	Intelligibility with familiar people, less familiar people and strangers Intelligibility in a stressful, emotional, or high-pressure situation Intelligibility in one-to-one settings, group settings and phone conversations.
2. Ability to participate in conversations	Starting, ending, sustaining, and contributing to conversations Conversations with familiar people, less familiar people, and strangers Conversations in one-to-one settings, group settings and phone conversations.
3. Living well with dysarthria	Comfortable and confident in everyday communication, individual or group settings Emotional and psychological well-being Participating in everyday life and social interactions, friendships/work/occupation whether in groups or with individuals
Where relevant to clinical practice and to research studies focussed on communication partners this 4^th^ core outcome should be considered:	
4. Communication partner skills and knowledge	Equal responsibility for conversation breakdown including interruptions. Pays attention to individual and the content of conversation (inclusion and engagement). Understands challenges of dysarthria.

## Discussion

Through rigorous consensus work with key stakeholder groups, including those with lived experience, we have successfully agreed on what domains should be measured for dysarthria after stroke, completing the first step of the development of a core outcome set. Having this agreed set of domains for consideration in clinical practice and research should help to improve reporting of outcomes for people with dysarthria^5,7^ and help researchers to compare and collate findings so we can evaluate treatments more easily.^7^ We have consensus that the core outcome set for speech after stroke should include the following four topics as critically important to be measured: (1) intelligibility of speech, (2) ability to participate in conversations, (3) living well with dysarthria, (4) (where relevant) skills and knowledge of communication partners.

Intelligibility, which we defined in our survey questions as ‘can a person be easily understood by others’, had high levels of agreement across all stakeholder groups. Intelligibility is a continuum, where people may be more or less intelligible depending on intrinsic and extraneous factors as well as context and environment.^17^ It was clear from our discussions and the survey findings that our participants agreed intelligibility is best judged on everyday communications and interactions. It was also deemed important that the views of the individual with dysarthria, the speech and language therapist/pathologist and family/friends were considered in measuring intelligibility. There are well-documented challenges around how speech is perceived and understood by others, which is an issue to consider when we explore what measurement tool is best placed to measure intelligibility in everyday interactions.^17–19^

Participating in conversations was critically important. All aspects of conversation were considered critical, this included starting, sustaining, contributing to, and ending a conversation. This included conversations in a variety of contexts including group, telephone and noisy or quieter environments. There were three distinct groups, (1) people known very well and seen daily or often such as family and close friends, (2) people known fairly well and seen fairly often weekly or monthly and (3) strangers. We know how important social connections are, particularly for stroke survivors and the associated high risk of depression.^20^ This supports the importance of conversation ability in everyday communication as critically important to be measured for people with dysarthria after stroke.

The living well with dysarthria domain highlighted the importance of quality of life, psychological well-being and confidence in communication. Communication impairment contributes to an adverse impact on quality of life^21^ and this was recognised by all stakeholder groups. It was recognised in our consensus meeting discussion that this outcome also involved dealing with the perception of others and broader societal reactions to people with ‘different’ sounding speech. It also related to intrinsic factors of the individual with how confident they felt about their speech and how this change is an important factor in adjusting to life after stroke.

The inclusion of the outcome relating to conversation partners led to some useful discussion with clinical colleagues. They suggested the ability of communication partners was often a key goal of stroke survivors to ensure that family and friends improved their skills. Our discussion led to an agreement in the meeting that this would be a key outcome for clinical practice and could be used in relevant research where for example the research involved training communication partners. There is evidence of benefits for communication partner training for people with aphasia but further research is needed in the dysarthria population, and consideration given to what measurement tools may be relevant for this population.^22^

It became apparent from this Delphi process that activity and participation level outcome domains were considered most important by all three stakeholder groups, more so than impairment level outcomes. This is in contrast to the majority of traditional outcomes seen in previous dysarthria research^4^ and clinical practice.^23^ This may reflect the existing measurement instruments for dysarthria which are often impairment focussed. To finalise a core outcome set for dysarthria further work needs to establish what measures are suitable for the outcomes we have identified and whether new measures need to be developed.

Limitations of the study related to challenges with recruitment, particularly for stroke survivors. We had limited success with recruiting people through online social media adverts and improved our recruitment by in-person attendance at stroke groups locally.^24^ This limited our pool of potential stroke survivors from a national focus to a regional one. Attending local groups however, did allow us to recruit a small number of people (*n* = 3, 11%–17% of the stroke survivors recruited) from a diverse background when 8% of the UK stroke population is from a black, Asian, or other ethnic groups (84% white background, 8% not stated/unknown).^25^ We know globally there is a disparity in stroke risk when comparing white with other ethnic groups, who have earlier stroke onset and worse outcomes.^26^

Core outcome set development must include those with lived experience, to ensure research and clinical practice measures outcomes relevant to the population affected.^27^ Integral to the research team were our three stroke survivors who have lived experience of dysarthria and their involvement shaped the study, changed our thinking and improved this research project.^28^ Our learning from the consensus meetings suggests that carrying out COS work with stroke survivors benefits from accessible materials, debrief opportunities, live support via phone or text during meetings, shorter meetings with breaks and opportunity to listen and discuss.

This consensus work to agree on what outcomes should be measured for dysarthria after stroke was carried out in the UK and Australia only. ******This is a limitation of the study, but was a pragmatic decision to reflect the funding and timeframe to support this work. Another limitation was that we did not collect demographic data from the participants who were therapists or researchers. The next steps with this work will be to identify existing measurement tools for dysarthria and match these to the outcome domains where possible. Agreeing ‘how’ to measure these outcome domains will complete this core outcome set for dysarthria and bring it in line with the core outcome set for aphasia.^9^ Implementation of core outcome sets are more likely to be used when measurement tools are included with the core outcome set.^10,11^ Dissemination will promote these domains of ‘what’ to measure but it may be that other countries wish to investigate this further with their own stroke survivor and clinician population^11,29^ to encourage use. We continue to report our findings in an accessible way and have published a blog https://www.evidentlycochrane.net/stroke-survivors-measuring-what-is-important-in-speech-recovery/.

**Clinical messages**

- Clinicians should use these core outcome domains to guide goal setting and treatment for dysarthria after stroke.
- Therapy must be evaluated by whether it successfully achieves outcomes that are valued by all stakeholders including people with dysarthria.
- Until a core outcome set is finalised clinicians and researchers should choose measures that map onto the outcome domains agreed in this study.
